# Familial hemiplegic migraine due to CACNA1A and PNKD mutations in epilepsy with forced normalization: A case report

**DOI:** 10.1097/MD.0000000000041844

**Published:** 2025-08-15

**Authors:** Aleida Arritola-Uriarte, Daniel San-Juan, Lenin V. Sandoval-Luna, David J. Anschel, Antonio Guechi

**Affiliations:** a Epilepsy Clinic, National Institute of Neurology and Neurosurgery, Mexico City, Mexico; b Epilepsy Clinic, St. Charles, Port Jefferson, NY; c New York Institute of Technology, New York University School of Medicine, Old Westbury, NY; d Institute of Molecular Biology in Medicine and Gene Therapy, University Center for Health Sciences, University of Guadalajara, Jalisco, Mexico.

**Keywords:** CACNA1A gene, epilepsy, family hemiplegic migraine, force normalization, migraine, migralepsy

## Abstract

**Rationale:**

Hemiplegic migraine (HM) is a rare subtype of migraine characterized by complex aura and transient hemiparesis. It is infrequently associated with refractory focal epilepsy, and there are no previous reports of forced normalization (FN) in this context. This case highlights a novel clinical association and the diagnostic and therapeutic challenges it presents.

**Patient concerns:**

A 31-year-old right-handed woman presented with episodes of cognitive impairment following seizure control, as well as recurrent episodes of HM and prolonged focal seizures. She had a history of familial HM associated with *CACNA1A* and *PNKD* mutations.

**Diagnoses:**

Genetic testing confirmed the presence of *CACNA1A* and *PNKD* mutations, consistent with familial HM. The patient was also diagnosed with focal refractory epilepsy and exhibited clinical and electroencephalographic features suggestive of FN.

**Interventions:**

The patient received various antiseizure medications, with adjustment of dosages and regimens in response to status epilepticus and evolving cognitive symptoms. Treatment was tailored to balance seizure control while minimizing adverse neuropsychiatric effects.

**Outcomes:**

Seizure control was partially achieved with adjustment of antiseizure medications; however, episodes of cognitive dysfunction persisted during electroencephalogram normalization periods, consistent with FN. Functional status improved gradually with individualized treatment, but neurological deficits and migraine persisted intermittently.

**Lessons:**

This case illustrates a rare coexistence of familial HM, focal refractory epilepsy, and FN. It emphasizes the need for heightened clinical awareness of FN in similar complex neurogenetic disorders and highlights the importance of individualized pharmacological strategies.

## 1. Introduction

Hemiplegic migraine (HM) is a rare subtype of migraine characterized by complex aura symptoms and motor impairment, commonly unilateral weakness. Onset is typically in the first or second decade of life, and females are 2.5 to 4 times more affected than males^.[1,2]^ The prevalence of HM was estimated to be 0.01% in Denmark.^[3]^ HM can be present as sporadic hemiplegic migraine in the 0.002%, whereas familial hemiplegic migraine (FHM) can be found in the 0.003% of all cases,^[3]^ in which there is at least one first- or second-degree relative with similar symptoms. Four associated genes have been identified: calcium voltage-gated channel subunit alpha 1A (CACNA1A), ATPase Na+/K+ transporting subunit alpha 2 (ATP1A2), sodium voltage-gated channel alpha subunit 1 and proline rich transmembrane protein 2.^[1,2]^ The coexistence with epilepsy accounts for 40% of overall HM patients, and drug-resistant forms of epilepsy are rare in all FHM mutations.^[4]^

Forced normalization (FN) is the disappearance of epileptiform activity in the electroencephalogram (EEG) and seizure control in patients with epilepsy, followed by severe acute behavioral changes.^[5]^ It has been described as occurring in 7.8% of adults with epilepsy.^[6]^ It may have a higher frequency in women (61.5%).^[5]^ There is no documented presence of FN in patients with FHM and refractory epilepsy. We present a unique and complex patient with HM and FN.

## 2. Case report

A 31-year-old right-handed female sought medical attention for new-onset refractory severe psychosis. Her family history revealed a maternal grandmother with epilepsy and migraines. She had a mild intellectual disability due to neonatal hypoxia caused by umbilical cord compression during vaginal delivery. A pivotal event occurred at 18 months when a moderate traumatic brain injury triggered her first generalized tonic-clonic seizure. Subsequently she developed focal seizures, documented by scalp EEG, characterized by visual auras with many colors without much brightness on the right periphery of the visual field with a subsequent 5-minute episode of severe right pulsating hemicranial headache (10/10 by visual analogue score) with nausea and vomiting and left hemiparesis, lasting 12 to 24 hours. The events never resulted in loss of consciousness, and there was a poor response to valproic acid and carbamazepine. Clusters of these episodes led to multiple hospitalizations.

In November 2007, at the age of 15, the patient presented to the emergency department with a severe right pulsating hemi cranial headache, accompanied by acute left facial and body (3/5) hemiparesis and bilateral ataxia. Her blood tests revealed leukocytosis and neutrophilia. Head computed tomography scan and 1.5 T brain magnetic resonance imaging were normal; brain single photon emission computed tomography showed right hemispheric hypoperfusion, and a standard scalp EEG exhibited right hemisphere slowing and epileptiform discharges in the right frontal and occipital regions. A lumbar puncture was normal, and cultures were negative. She received topiramate as add-on therapy and was discharged without complications or neurological deficits after 7 days. At age 20, in April 2012, there was another HM episode that led to worsening neurological conditions that needed mechanical ventilation and admission to neurocritical care. A non-convulsive status epilepticus was diagnosed and treated with midazolam, but no new neuroimaging findings were found. Abnormal EEG and symptoms resolved after 5 days. In 2016, at the age of 24, a new HM event prompted hospitalization for a complex partial status with a scalp EEG showing 3 Hz right hemispheric slowing and right fronto-temporal epileptiform activity. She was treated with mechanical ventilation, levetiracetam, valproate, and midazolam with resolution at 7 days.

**Figure 1.**
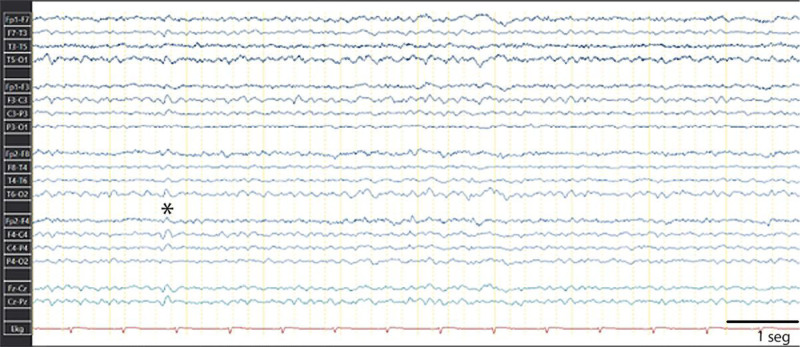
Awake 20-min, standard scalp EEG showing a generalized 6 to 7 Hz slowing and rare right fronto-temporal interictal epileptiform discharges (*). Filters 1 to 70 Hz; notch+; sensitivity 7 µV/mm. EEG = electroencephalogram.

In 2019, at the age of 27, and after 3 years of follow-up without any symptoms or paroxysmal neurological events, the patient received a diagnosis of FHM secondary to CACNA1A and PKNPD gene mutations and started lacosamide (400 mg/d) as an add-on to topiramate (100 mg/d) with good seizure control and paroxetine (20 mg/d) and ketorolac for pain. By 2020, parents noted a cognitive decline (10/30 Mini Mental Status Examination of Folstein) and severe psychosis with visual hallucinations, soliloquy, and verbal aggressiveness towards them intensifying in the evening, initially treated during 2 years with quetiapine (50 mg/d) and risperidone (4 mg/d) without improvement. She was diagnosed with forced normalization (Table 1) and started on October 2023, with verapamil 40 mg “bis in die”/twice a day (BID), paroxetine 2quarterquater in die”/ 4 times a day, withdrawal of topiramate and decrease of lacosamide 100 mg BID, and started with clozapine 50-0-100 mg BID with recovery of the baseline cognition (27/30 Mini Mental Status Examination of Folstein) and mild and brief episodes of psychosis with occasional or mild visual hallucinations that do not significantly impair daily functioning. A new scalp standard EEG shows generalized 6 to 7 Hz slowing and occasional right fronto-temporal interictal epileptiform discharges (Fig. 1).

**Table 1:** Forced normalization diagnostic criteria.

Primary (essential) criteria
1. Established diagnosis of epilepsy based on clinical history, EEG, an imaging 2. Presence of a behavioral disturbance of acute/subacute onset characterized by 1 or more of the following: Psychosis with thought disorder, delusions, hallucinations Significant mood change, hypomania, mania or depression Anxiety with depersonalization, derealization 3A. Reduction in the total number of spikes counted in 60 min awake EEG recording with a 16-channel machine, using standard 10 to 20 electrode placement, by over 50% compared to a similar recording performed during a normal state of behavior. 3B. Report of complete cessation of seizures for a least 1 wk corroborated any relative or caregivers.
Supportive criteria
Recent change (within 30 d) of pharmacotherapeutic regimen Report of similar episodes of seizure cessation and behavioral disturbance in the past, from close relative or caregivers, or general practitioner, or documentation of this in hospital records with or without EEG evidence. This may or may not be linked with and anticonvulsant drug
To make the diagnosis
Primary criteria 1, 2 and 3A OR Primary criteria 1, 2 and 3B and 1 supportive criterion

EEG = electroencephalogram.

## 3. Discussion

To our knowledge, this is the first report of a patient with FHM and focal refractory epilepsy with FN after the use of antiseizure medications (ASMs) with partial resolution. Our FHM patient displayed a head trauma trigger and the clinical manifestations previously described,^[1]^ however, developed a non-classical episode of FN^[5]^ with a seizure-free period of 6 years. Nonetheless, on the follow-up, she developed severe and refractory chronic psychosis. FN triggers include the use of ASMs or epilepsy surgery, which can manifest as psychosis.^[7]^ A 2019 review of 65 patients with epilepsy and FN found that the most common trigger factor was the use of ASMs (48.5%), mainly levetiracetam (25%), or topiramate (3.1%), followed by epilepsy surgery (31%).^[7]^ Specifically, in patients with CACNA1A-associated epilepsy, topiramate has demonstrated encouraging effectiveness at seizure control.^[8]^ Carazo Barrios et al^[9]^ describe a case series of 10 patients with FN and drug resistance epilepsy, in which the FN treatment was mainly based on the use of antipsychotic medication (mostly quetiapine), antidepressants, and lowering the number of suspicious ASMs.

In the follow-up, 7/10 patients returned to baseline, 2 showed partial improvement of the behavioral symptoms, and 1 patient had no improvement. Unfortunately, 4/10 patients showed recurrence of seizures weeks or months after improvement of the psychiatric symptoms.^[9]^ In our patient, we decreased the doses of lacosamide, withdrew topiramate, and started clozapine up to 150 mg/d. A single case report, involving a 33-year-old female, linked topiramate intake to an exacerbation of symptoms in sporadic HM.^[10]^ Then, she improved considerably, decreasing the frequency of chronic psychotic symptoms and improving her cognition, which confirmed the reversible nature of the neurological entity. In our patient, topiramate and lacosamide might have triggered factors to develop FN.

The physiopathology of FN is unknown; however, some of the neurotransmitters implicated with the mechanism of action of ASMs related to FN include dopamine, glutamate, and GABA, supporting the theory of GABAergic facilitation leading to an increase in dopamine activity^.[7]^ Genetically, our patient has a unique combination of 2 genetic mutations associated with FHM: CACNA1A and Paroxysmal nonkinesigenic dyskinesia (PNKD) gene (Table 2), where a novel PNKD gene deletion has been identified in a family with isolated HM, indicating a potential genetic overlap between these conditions.^[11]^ Researchers have extensively studied the CACNA1A gene in the context of FHM, locating it at 19p13.^[12]^ This mutation is not only linked to FHM but also to several other neurological diseases, such as episodic ataxia type 2, spinocerebellar ataxia type 6, Lennox–Gastaut syndrome, Dravet syndrome, and autism spectrum disorder.^[12]^ It is hypothesized that CACNA1A mutations related to HM tend to involve insertions or deletions, which provoke frameshifts within the open reading frame, resulting in missense mutations, affecting functional domains of calcium channel, resulting in gain-of- function effects and increasing Ca^2+^ influx and enhanced glutamate release at cortical synapses. However, the opposite effect in Ca_2_ channels influx has been found as well.^[12]^ The inclusion of the PNKD gene mutations adds an additional layer of complexity to this case. Rarely, patients with FHM have observed its association with paroxysmal non-kinesigenic dyskinesia. PNKD interacts with synaptic active zone proteins, and the mutant protein is less effective at inhibiting exocytosis, resulting in increased neurotransmitter release^.[1,11]^

**Table 2:** Report of the genetic test evaluating 146 genes for variants associated with genetic disorders, with 5 variants of uncertain significance.

Gene	Protein	Variant	Zygosity
CACNA1A	Calcium voltage-gated channel subunit alpha 1A	Exon 36 c.5430A > G p.His1827Arg ClinVar: 860639	Heterozygous
DOCK7	Dedicator of cytokinesis 7	Exon 25 c.2993G > A p.Val1000Ile ClinVar: 860640	Heterozygous
PNKD	Paroxysmal non-kinesigenic dyskinesia metallo-beta-lactamase domain containing	Exon 6 c.559C > T p.Arg187Trp ClinVar: 468634	Heterozygous
PRIMA1	Proline rich membrane anchor 1	Exon 3 c.146G > A p.Arg49Gln ClinVar: 860641	Heterozygous
TBC1D24	TBC1 domain family member 24	Exon 2 c.809G > A p.Arg270His ClinVar: not available	Heterozygous

FHM was classified into 4 subtypes (FHM 1–4) according to the mutation associated with a specific gene: CACNA1A; ATP1A2; and sodium voltage-gated channel alpha subunit 1.^[2,13]^ FHM subtype 4 is diagnosed when no known genetic mutation is linked to FHM.^[13]^ Importantly, the association between HM and epilepsy is not uncommon, with approximately 40% of patients presenting with both conditions.^[4]^ However, the simultaneous presence of mutations in more than 1 gene, as observed in our patient, poses challenges for understanding the underlying mechanisms, clinical manifestations, and therapeutic management.

We followed the diagnostic approach, starting with a clinical evaluation based on the classification criteria for hemiplegic migraine, as presented in Table 3. We conducted additional ancillary studies, such as lumbar puncture and magnetic resonance imaging, to rule out differential diagnoses, both of which yielded normal results. Given the persistence of diagnostic suspicion, we proceeded with genetic analysis, which yielded positive results, thus correlating the clinical diagnosis.

**Table 3:** Diagnostic criteria for a hemiplegic migraine as per the International Classification of Headache Disorders-3.

Two attacks fulfilling the criteria fulfilling criteria for migraine with aura • Fully reversible motor weakness • Fully reversible visual, sensory, and/or speech/language symptoms
Criteria
A. At least 2 attacks that fulfill criteria B and C
B. One or more of the following aura symptoms that are reversible: visual, retinal, sensory, brainstem, motor, speech, or language
C. At least 3 of the 6 characteristics below: ■ At least 1 aura symptom that spreads gradually over >5 min ■ Two or more symptoms in succession ■ At least 1 unilateral aura symptom ■ At least 1 positive aura symptom ■ Each aura symptom lasting 5 to 60 min ■ Aura accompanied by or followed by headache within 60 min
D. No other ICHD-3 diagnosis accounting for the symptoms

The current management of HM is empirical due to the lack of randomized controlled studies. However, single-case reports and patient series (1–76 patients) reported the use of triptans, nimodipine, calcitonin gene-related peptide (CGRP), ketamine, corticosteroid pulses, naloxone, and furosemide in acute cases, which generally demonstrated benign evolution and control over the intensity and frequency of cephalalgia episodes.^[1]^ Prophylactic treatments recommended included verapamil, acetazolamide, flunarizine, lamotrigine, propranolol, memantine, telcagepant, topiramate, and onabotulinumtoxin A.^[1]^ Topiramate was the first treatment given to our patient. Due to FN, we removed topiramate, an ASM with multiple mechanism of actions including, a voltage-gated sodium and voltage-gated calcium channels antagonist, inhibitor of α-amino-3-hydroxy-5-methyl-4-isoxazolepropionic acid (AMPA) and kainate glutamate receptors, increase the effects of gamma-aminobutyric acid (GABA) A receptors, and inhibitor of carbonic anhydrase from the therapeutical schema in the last follow-up and replaced it with verapamil, L-type calcium channel antagonist, resulting in no new episodes of HM until the last follow-up, however, this have a limited generalizability to clinical practice and evidence from other patients and clinical trials are needed. One case reported that after a patient switched to topiramate, their seizures stopped and epileptiform discharges on the EEG were reduced, but severe abnormal mental behavior appeared. These mental abnormalities disappeared after stopping the drug and reappeared after reintroducing topiramate.^[14]^ We achieved the goal of returning the patient to baseline, restoring normal functioning without psychosis, while preventing a new episode of status epilepticus and HM.

## 4. Conclusion

In conclusion, we present a unique and intricate case involving a patient with HM experiencing refractory epilepsy and forced normalization due to ASM, with a balanced and complete resolution. While the symptomatic improvement was achieved, it underscores the necessity for a multidisciplinary approach and ongoing monitoring to optimize clinical outcomes in the long term. This case also highlights the importance of further elucidating the underlying mechanisms of epilepsy in HM patients, as well as developing more effective therapeutic strategies to address this complex medical condition.

## Author contributions

**Conceptualization:** Daniel San-Juan.

**Data curation:** Antonio Guechi.

**Investigation:** Daniel San-Juan, Lenin V. Sandoval-Luna, Aleida Arritola-Uriarte.

**Methodology:** Daniel San-Juan, Lenin V. Sandoval-Luna, Aleida Arritola-Uriarte.

**Project administration:** Daniel San-Juan.

**Supervision:** Daniel San-Juan.

**Validation:** Daniel San-Juan.

**Visualization:** Daniel San-Juan.

**Writing – original draft:** Daniel San-Juan, Lenin V. Sandoval-Luna, Aleida Arritola-Uriarte, David J. Anschel, Antonio Guechi.

**Writing – review & editing:** Daniel San-Juan, Lenin V. Sandoval-Luna, Aleida Arritola-Uriarte, David J. Anschel, Antonio Guechi.
